# The load carriage index (LCI) - adjusting the load carried by the soldier according to body composition measurements

**DOI:** 10.1186/2046-7648-4-S1-A10

**Published:** 2015-09-14

**Authors:** Itay Ketko, Amir Hadid, Ran Yanovich, Yoram Epstein, Yuval Heled

**Affiliations:** 1 The Warrior Health Research Institute, Medical Corps, Israel Defence Forces. Heller Institute of Medical Research, Sheba Medical Center, Tel Hashomer, Israel. Department of Biomedical Engineering, Faculty of Engineering, Tel Aviv University, Israel. Sackler Faculty of Medicine, Tel Aviv University, Israel

## Introduction

Lean body mass (LBM), strongly correlates with absolute maximal oxygen uptake (VO_2_max), has been shown to predict load carriage performance [1]. In contrast, fat mass is considered "dead mass" (DM) that reduces mobility and effectiveness of the carrier[2,3]. Lyons et al. proposed that the ratio LBM to DM can indicate the ability to carry loads [3]. We aimed to study the ability to better distribute the loads to be carried by a team of soldiers by using a load carriage index (LCI) rather than relying only on percentage of bodyweight.

## Methods

The load carriage Index was applied as follows: LCI = (lean body mass)/(fat mass + external load) 14 healthy males (age: 26(2) yrs; weight: 77(12) kg; fat percentage: 17(4) %; VO_2max_: 52.1(5.6) mL.kg^-1^.min^-1^) performed light exercise on a motor driven treadmill (4 km.h^-1 ^and 0% incline), while carrying 40% of their bodyweight (BW). The LCI was calculated for each subject according to his anthropometric measures and the load he carried. The oxygen consumption was measured continuously during the exercise.

## Results

The average load weight carried by the subjects was 31(5) kg, which corresponded to an average LCI of 1.46(0.16) (range of 1.21-1.70). The metabolic demand (%VO_2max_) was 26.7(3.4) %. A strong correlation with LCI (Spearman p = -0.68, p < 0.006), %Fat (Spearman p of 0.70, p < 0.005) and relative VO_2_max (Spearman p of -0.89, p < 0.001.) The ability of the LCI to enable a better distribution of the loads within the study group is exemplified by the following (table 1): the two subjects with similar weight and VO_2_max were required to carry almost the same load (~30 kg). The LCI was lower for the subject with the higher %body-fat, resulting with a higher metabolic demand (%VO_2max_). By matching LCI for both subjects, without changing the total weight to be carried, almost 5 kg could be shifted from subject #1 to subject #2.

**Table 1:** 

Subject	BW [kg]	Fat [%]	LBM [kg]	Load [kg] (40% BW)	LCI	VO_2_max [mL/kg/min]	%VO_2_max	New LCI	New Load [kg]	New Load [%BW]
**#1**	73	19.7	58.6	29.2	1.35	60.0	25.5	1.51	24.5	33.5

**#2**	75	12.0	66.0	30.0	1.69	61.0	17.6	1.51	34.7	46.3

## Discussion

LCI varies considerably within the group while requiring carrying the same %BW. This is due to the higher DM carried by those with the higher %body-fat. Thus, in order to match work intensity (similar metabolic demand of the task) between different individuals carrying loads we suggest the LCI as a helpful index for a better given load distribution, rather than relying only on percentage of body mass only.
